# Editorial: Exploring the interconnection: obesity's role in asthma development and management

**DOI:** 10.3389/falgy.2025.1759789

**Published:** 2026-01-21

**Authors:** Akira Yamasaki, Katsuyuki Tomita

**Affiliations:** 1 Division of Respiratory Medicine and Rheumatology, Department of Multidisciplinary Internal Medicine, School of Medicine, Faculty of Medicine, Tottori University, Yonago, Japan; 2 Department of Allergy and Clinical Immunology, Hakuai Hospital, Yonago, Japan

**Keywords:** commorbidity, corticosteroid insensitivity, gut microbiome, obesity, type2 low inflammation

A comprehensive study on the association between obesity and asthma is urgently needed owing to the global increase in the prevalence of both conditions. Owing to similar inflammatory processes and immunological dysfunction, obesity worsens asthma symptoms and increases the risk of exacerbation. The scope of this volume of obesity-associated asthma covers epidemiological assessments of comorbidities, inflammation, and immunological responses that link obesity to asthma, corticosteroid insensitivity, and the effects of obesity-related metabolic dysfunction on asthmatic airway remodeling.

Zhang et al. reported that the death and disability-adjusted life year rates of asthma are attributable to a high body mass index among children and adolescents, with varying trends across different Socio-Demographic Index regions and sex groups extracted from the Global Burden of Disease from 1990 to 2021.

Shailesh et al. investigated the link between elevated serum levels of pro-inflammatory markers, such as IL-2, IL-5, IL-33, and TNF-α, and obesity-associated asthma in children without a synergistic or cumulative influence on inflammatory response. According to their research, children with asthma and overweight/obesity can be successfully treated with therapeutic approaches, such as steroid therapy or biologics.

Based on the 2007–2020 National Health and Nutrition Examination Survey, Ma et al.'s epidemiological study on children and adolescents reported that the prevalence of hypertension was 6.0% for asthma, 9.4% for obesity, and 12.8% for asthma and obesity, with a synergistic impact. After a stratified subgroup analysis based on age, sex, birth weight, and tobacco exposure, they observed that the risk remained substantial, underscoring the need for focused treatment in overweight children and adolescents.

Tashiro et al. focused on the existence of complex and multilayered crosstalk between the bidirectional gut–lung axis and the host immune system through bile acids, short-chain fatty acids, and glucagon-like peptide 1 as an interaction between obesity and asthma. This review proposes potential treatments for obesity-associated asthma that target the gut microbiome.

To and To addressed the possibility of corticosteroid resistance in patients with obesity and asthma. They proposed several possible causes of corticosteroid insensitivity in patients with obesity and asthma, including a decreased GRα/GRβ ratio, higher Th17/IL-17 responses, increased leptin secretion, attenuated AMPK and SIRT1 expression and activity, and increased systemic oxidative stress.

Listyoko et al. provided an overview of the three phases of airway remodeling in patients with obesity and asthma. Obesity-associated airway epithelial remodeling may involve various factors, including HMGB1, leptin, CysLTs, ORMDL3, matrikines such as PGP, and cytokines such as IL-4 and IFN-γ. Obesity-associated airway smooth muscle remodeling is an intricate process influenced by factors such as neutrophils, neutrophil elastase, CD4+ T cells, HMGB1 protein, TGF-β1, leukotrienes, and their derivatives. Obesity-associated bronchial vascular airway remodeling is influenced by factors such as VEGF, leptin, adiponectin deficiency, and angiopoietin.

**Figure 1:**
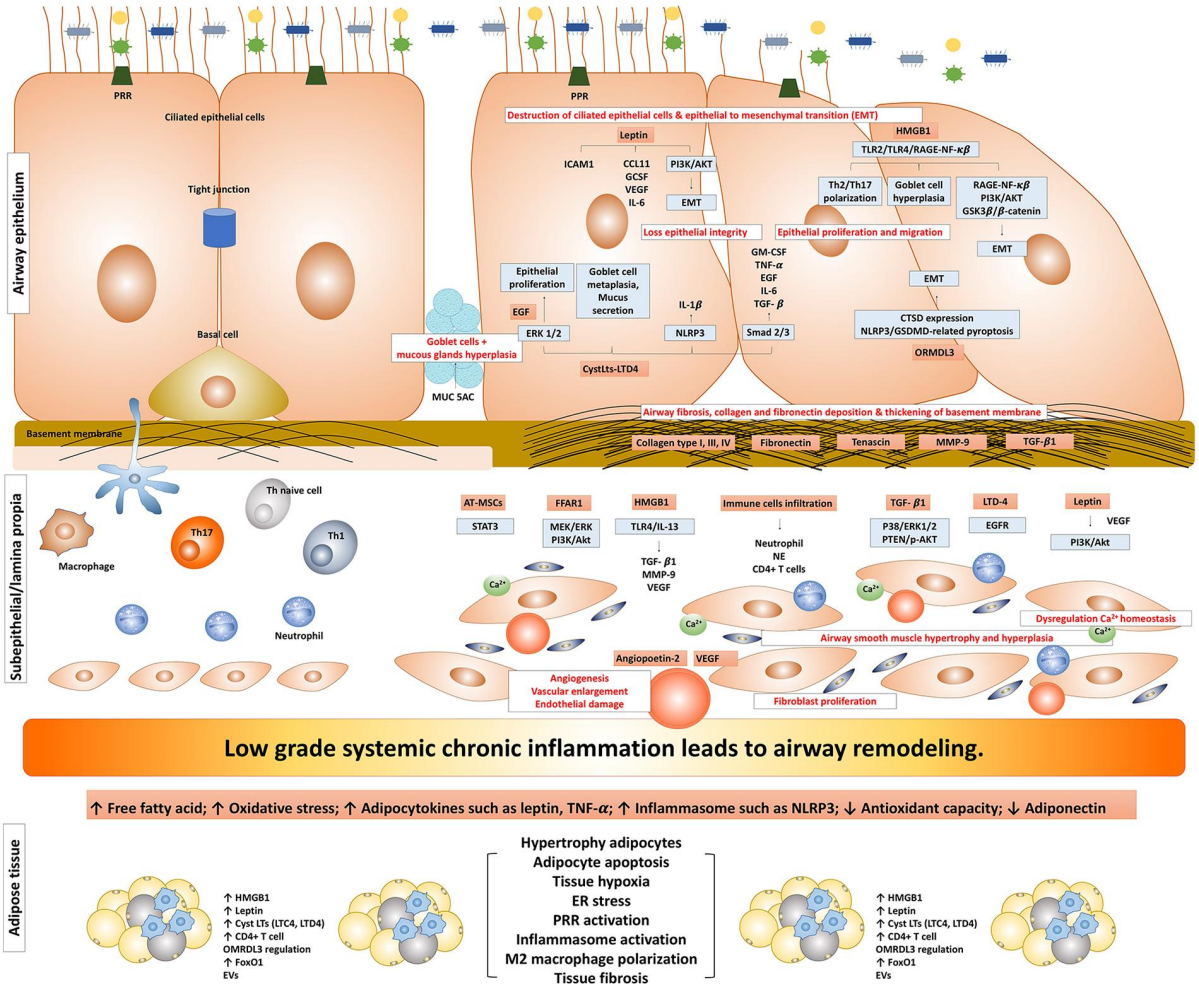
Possible mechanisms of obesity-induced airway remodeling in patients with obesity and asthma. Reproduced from “The possible mechanism of obesity-induced airway remodeling in obese-asthma” by Aditya Sri Listyoko, Ryota Okazaki, Tomoya Harada, Genki Inui and Akira Yamasaki, licensed under CC BY 4.0.

We are confident that these articles will offer readers unique perspectives and useful information on a subject that necessitates further understanding of type2-low and neutrophilic inflammation as a low-grade systemic chronic inflammation in asthma with obesity from the perspective of the heterogeneous pathophysiology of asthma (Figure 1).

